# From pain to tumor immunity: influence of peripheral sensory neurons in cancer

**DOI:** 10.3389/fimmu.2024.1335387

**Published:** 2024-02-16

**Authors:** Ugo Mardelle, Ninon Bretaud, Clara Daher, Vincent Feuillet

**Affiliations:** Aix-Marseille Université, Centre National de la Recherche Scientifique (CNRS), Institut National de la Santé et de la Recherche Médicale (INSERM), CIML, Centre d’Immunologie de Marseille-Luminy, Marseille, France

**Keywords:** peripheral sensory neurons, nociceptors, neuropeptides, pain, cancer, tumor microenvironment, immune cells, tumor immunity

## Abstract

The nervous and immune systems are the primary sensory interfaces of the body, allowing it to recognize, process, and respond to various stimuli from both the external and internal environment. These systems work in concert through various mechanisms of neuro-immune crosstalk to detect threats, provide defense against pathogens, and maintain or restore homeostasis, but can also contribute to the development of diseases. Among peripheral sensory neurons (PSNs), nociceptive PSNs are of particular interest. They possess a remarkable capability to detect noxious stimuli in the periphery and transmit this information to the brain, resulting in the perception of pain and the activation of adaptive responses. Pain is an early symptom of cancer, often leading to its diagnosis, but it is also a major source of distress for patients as the disease progresses. In this review, we aim to provide an overview of the mechanisms within tumors that are likely to induce cancer pain, exploring a range of factors from etiological elements to cellular and molecular mediators. In addition to transmitting sensory information to the central nervous system, PSNs are also capable, when activated, to produce and release neuropeptides (e.g., CGRP and SP) from their peripheral terminals. These neuropeptides have been shown to modulate immunity in cases of inflammation, infection, and cancer. PSNs, often found within solid tumors, are likely to play a significant role in the tumor microenvironment, potentially influencing both tumor growth and anti-tumor immune responses. In this review, we discuss the current state of knowledge about the degree of sensory innervation in tumors. We also seek to understand whether and how PSNs may influence the tumor growth and associated anti-tumor immunity in different mouse models of cancer. Finally, we discuss the extent to which the tumor is able to influence the development and functions of the PSNs that innervate it.

## 1 Introduction

A tumor is more than a mere collection of cells but a complex ecosystem known as the tumor microenvironment (TME), encompassing cells and soluble factors that play a pivotal role in tumor growth (1). Among these constituents, immune cells hold undeniable importance, as evidenced by the advent of immunotherapy (2). Nonetheless, despite unprecedented clinical benefit in various cancers, only a minority of patients respond to current immunotherapies. Therefore, it is crucial to explore the mechanisms within the TME impacting the development of an effective anti-tumor immune response either natural or therapeutically induced.

Nerve fibers in solid human tumors, often associated with a poor prognosis, have long been underappreciated for their potential influence on tumor progression and, more specifically, on the anti-tumor immune response (3). Among them, peripheral sensory neurons (PSNs) and, more specifically, nociceptive PSNs known as nociceptors innervate peripheral tissues and elicit painful sensations after sensitization of their terminals by noxious signals. They possess multiple receptors allowing the detection of pathogen-associated molecular patterns (PAMPs), damage-associated molecular patterns (DAMPs), and immune mediators during tissue injury or infection. Additionally, nociceptors release neuropeptides, including calcitonin gene-related peptide (CGRP) and substance P (SP), capable of modulating the functions of immune cells, which express the corresponding neuropeptide receptors (4). In the past decade, studies have revealed interactions between nociceptors and the immune system, impacting pain, inflammation, and host defense regulation. Specifically, nociceptors can modulate antimicrobial or inflammatory immune responses and contribute to chronic inflammatory disease pathogenesis (5–16). Furthermore, these studies have highlighted different capacities among nociceptor subsets, such as pro- or anti-inflammatory roles, in influencing the immune response (17).

While the influence of sympathetic and parasympathetic nerve fibers on tumor growth and anti-tumor immunity has been well-documented (18), PSNs have received relatively little attention in this context. Although a few studies have hinted at their potential role in tumor progression, apart from a few studies, their precise regulatory impact on the anti-tumor immune response remains poorly understood. We will present and thoroughly discuss these issues in this review. However, before delving into the details, considering our emphasis on nociceptors, we will first “set the stage” by addressing some basic questions about cancer pain.

Is cancer painful, and is pain a consistent feature across all cancer types? Although this question seems to be a simple one, the intricate nature of pain in various cancer types necessitates a clear definition of cancer pain and of the mechanisms underlying its triggering and perception. In this discussion, we will explore some pain-related mechanisms and with a special focus on the TME-induced signals that can activate nociceptors. We will also examine the extent of sensory innervation within tumors and its reciprocal relationship with carcinogenesis.

## 2 Cancer and pain

Given its heterogeneity, defining and treating cancer pain is a challenge, but a useful one. Indeed, pain is often the primary symptom prompting a cancer diagnosis but also a major source of patient distress during the disease’s progression.

### 2.1 What exactly is pain, and how is it generated?

PSNs form a family of neurons capable of detecting various stimuli in peripheral tissues. A significant proportion of these PSNs express the Nav1.8 sodium channel, including nociceptors/pruriceptors specialized in detecting and transmitting noxious stimuli that may induce pain. Historically, PSNs have been classified based on anatomical (e.g., fiber diameter, soma size, and localization), physiological (e.g., action potential velocities), and functional (e.g., neuropeptide production) properties. In addition, recent single-cell RNA sequencing studies have defined a comprehensive molecular and functional classification of PSN subsets in various species, including mice, macaques, and humans (19–23). Harmful stimuli provoking pain or itch are conveyed by different subsets of nociceptors/pruriceptors, which are further subdivided into peptidergic (PEP) or nonpeptidergic (NP), based on initial observation of their ability to produce canonical neuropeptides, SP and CGRP. Among PEPs, the PEP1 subgroup expresses Tac1, Calca, and Trpv1 (transcripts encoding SP, CGRP, and transient receptor potential vanilloid 1, respectively) and is therefore set to secrete CGRP and SP. Nociceptors are equipped with numerous receptors that enable them to detect perturbations in their environment. First, they express transient receptor potentials (TRPs), such as TRPV1, which are the main receptors involved in the detection and transduction of nociceptive stimuli. They also express receptors capable of recognizing various inflammatory or inflammation-associated mediators (microbial products, cytokines, neurotrophins, lipids and lipid-derived mediators, extracellular ATP, protons, etc.) produced by pathogens or cells. Finally, certain subtypes of nociceptors, including the C-low threshold mechanoreceptors (C-LTMRs) that express the Piezo2 receptor, can be excited by specific mechanical stimuli (24–26). The activation of these receptors by their ligands elicits their activation or increase in electrical activity, leading to hypersensitivity to external stimuli or amplification of pain perception and response.

**Figure 1:**
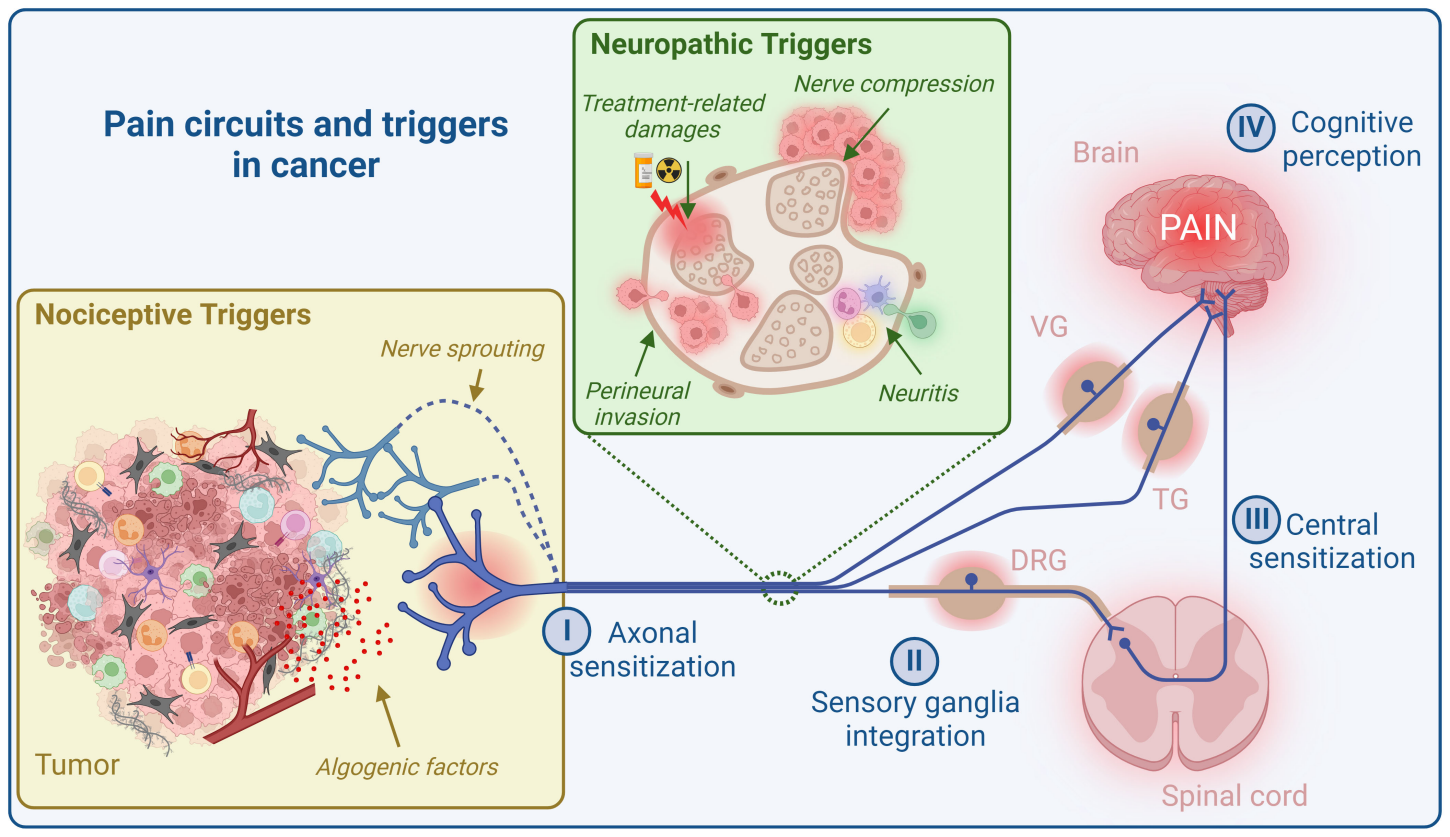
Pain circuit and its triggers in cancer. The cancer pain circuit comprises four levels of regulation. The initial level (I, axon sensitization) involves the sensitization of nerve endings of peripheral sensory neurons (PSNs) within the tumor microenvironment (TME), conveying neuronal information to sensory ganglia (DRG, VG, and TG) for integration (II, sensory ganglia integration). Next, PSNs from DRG afferent project nerve endings into the dorsal horn of the spinal cord, while PSNs from TG and VG project directly into the brainstem. In spinal cord and brainstem, neuropeptides (mainly SP, CGRP, and somatostatin) and glutamate from primary afferent fibers, along with other neurotransmitters such as gamma-aminobutyric acid (GABA) and glycine produced by second-order nociceptive neurons and interneurons, exert their effects on spinal and supraspinal neurons (III, central sensitization). Finally, spinal and supraspinal projection neurons relay this information to higher brain regions including the brainstem, somatosensory, insular, cingulate and prefrontal cortices, and thalamus and subcortical areas, where their integration can lead to pain perception (IV, cognitive perception). Cancer pain primarily arises from two main sources: “nociceptive triggers” and “neuropathic triggers”. Nociceptive stimuli encompass algogenic mediators released by the TME and nerve sprouting. Neuropathic triggers, on the other hand, are a consequence of nerve damage caused by tumor cell invasion (perineural invasion), immune cell invasion (neuritis), nerve compression, and the effects of radiotherapy and chemotherapy. DRG, dorsal root ganglia; VG, vagal ganglia; TG, trigeminal ganglia. Created with BioRender.com.

*What is the pain circuit?* There are at least four control levels that filter information for cognitive pain perception (**Figure 1**). First, thanks to muliple specific recetors, PSNs detect noxious signals in the periphery and convert this information into action potentials (I, axon sensitization). These signals then reach sensory ganglia, including dorsal root ganglia (DRG), vagal ganglia (VG), and trigeminal ganglia (TG), which house PSN cell bodies. These ganglia are also composed of other cell types, notably glial and immune cells, that collectively respond to incoming signals (27–29) (II, sensory ganglia integration). PSNs from DRG afferent project nerve endings into the dorsal horn of the of the spinal cord, while PSNs from TG and NG project directly into the brainstem. In the spinal cord and brainstem, neuropeptides (mainly SP, CGRP, and somatostatin) and glutamate from primary afferent fibers, along with other neurotransmitters such as gamma-aminobutyric acid (GABA) and glycine produced by second-order nociceptive neurons and interneurons, exert their effects on spinal and supraspinal neurons (30, 31) (III, central sensitization). Finally, spinal and supraspinal projection neurons relay this information to higher brain regions including the brainstem, somatosensory, insular, cingulate and prefrontal cortices, and thalamus and subcortical areas, where their integration can lead to pain perception (32–34) (IV, cognitive perception). Thus, this sequence of transmission and integration of sensory signals ultimately leads to conscious perception and appropriate behavioral responses (31, 35).

In the bidirectional communication between the brain and the body, a painful stimulus initially generates a danger signal that the brain perceives, subsequently triggering an adaptive response. The best illustration of this phenomenon is the withdrawal response, also known as the nociceptive flexion reflex, an automatic spinal cord reflex that plays a crucial role in protecting the organism against harmful stimuli perceived by nociceptors (36). In the case of acute inflammation, which is typically associated with pain, it can activate an anti-inflammatory reflex (37). However, a major problem arises when the stimulus becomes chronic, as in the case of chronic cancer pain. In such situations, the reflex that was suitable for an acute stimulus is unlikely to be suitable for one to a chronic, persistent one.

*Are there different types of pain?* There are indeed various pain types with different etiologies: nociceptive pain, inflammatory pain, neuropathic pain, and dysfunctional pain (38). Nociceptive pain may be viewed as a physiological pain due to the activation of high-threshold nociceptor neurons by noxious stimuli, not necessarily inflammatory ones. Inflammatory pain arises from inflammation in peripheral tissues, detected by nociceptors, leading to heightened sensitivity to pain. Neuropathic pain may result from somatosensory system damage or disease, altering nociceptive signal processing and leading to pain without stimuli or heightened responses to both innocuous and noxious stimuli. Dysfunctional pain occurs when there is no identifiable noxious stimulus, inflammation, or nervous system damage. More comprehensive and detailed information on these various types of pain, especially those involved in cancer, can be obtained from reviews specifically addressing these issues (31, 38–40).

### 2.2 Is a tumor always painful, and what is exactly cancer pain?

*Is a tumor painful?* Providing a concise response to this query is challenging due to numerous influencing factors. It hinges on the tumor’s characteristics, including its location, histological type, innervation level, immune cell infiltration, developpment stage/grade, and size. Additional factors, such as gender, psychological state, and genetic polymorphism, also play a role. As a result, pain tolerance varies from person to person.

However, it must be recalled that, often, cancer initially develops silently, without causing pain, making it difficult to diagnose. In addition, tumors are generally painless when small and only become painful or “unpleasant” when they grow, depending on their location. Therefore, it follows logically that cancer pain intensifies as the tumor grows and the disease advances. Thus, the prevalence of reported cancer pain varies primarily according to cancer stage, rather than cancer type (41). However, it is worth noting that the correlation between the level of pain and the tumor size is still debated, at least concerning oral cancers (42–44). Thus, in oral cancer, a report has been reported between tumor size and patient-reported pain and associated nociceptive behavior in a carcinogen mouse model (44), whereas such a correlation was not observed in other studies (42, 43). Moreover, although benign or precancerous lesions are generally painless, the transformation process may result in a sensation of pain. Thus, in a preclinical model of pancreatic cancer, it has been shown that neuroplastic changes occur in PSNs prior to transformation into cancer (45).

Certain types of cancer, notably oral, bone, and pancreatic cancer, are known to be highly painful (41, 46–49). In head and neck cancer (HNC), although the occurrence of pain is variable, almost all oral cancers are painful, and up to 85% of patients report pain at the time of diagnosis (46). In the case of bone, pain caused by bone metastases is the most frequent source of pain, and approximately 75% of patients with advanced cancer suffer from bone cancer pain (50). Moreover, systematic reviews have also indicated that the survival of advanced pancreatic cancer patients with cancer pain is notably shorter compared to those without (51). Because of their particularly painful nature, much of the knowledge about cancer pain discussed in this review comes from analysis of these cancer types.

*What causes cancer pain?* The etiology of cancer pain is multifaceted. Pain in cancer patients is believed to depend on factors such as the tumor’s mass, ulceration, inflammation, and infiltration. The underlying physiological mechanisms of cancer pain are usually viewed as a combination of inflammatory pain, arising from the release of numerous inflammatory factors within TME, and neuropathic pain, which results from damage to PSNs within the tumor, or during perineural invasion (PNI). However, a growing body of evidence suggests that cancer pain has unique characteristics that distinguish it from a simple combination of pain types already defined (39). This unique signature may explain the reduced efficacy of conventional analgesics (50).

Distinguishing between tumor-induced pain, treatment-related pain, and comorbid conditions is also important. Approximately 75% of pain is linked to the tumor itself, while 10%–20% results from treatments, especially chemotherapy and surgery, and the remaining 10% is attributed to comorbidities (52). For instance, cancer-related neuropathic pain can stem from treatment toxicity, vitamin deficiencies, tumor-induced nerve compression, or other problems such as diabetes and infection. Regardless of the cause, cancer pain becomes more prevalent as the disease advances, affecting approximately 64% of patients with advanced cancer (41, 49).

*Why are certain cancers more painful than others?* One obvious explanation is the level of tissue sensory innervation. For instance, the high degree of innervation of the oral cavity and bone tissue (53) may partly account for the heightened pain associated with cancers in these areas. However, this explanation does not hold entirely true, as melanoma, despite abundant skin innervation, is typically painless. The escalating frequency and intensity of pain as cancer progresses can be attributed to increased tissue innervation during tumor development. This occurs due to the remodeling of sensory and sympathetic nerve fibers through ectopic nerve sprouting (54–56). Additionally, differences in pain levels can be influenced by the histological nature and anatomical location of the cancer. Nevertheless, even within painful cancers, variations exist in pain phenotypes among histologically identical cancers at similar anatomical sites. These differences can be attributed to various factors, including etiology. For example, in the case of oropharyngeal squamous cell carcinoma, within the context of the same anatomical site, it has been shown that human papillomavirus (HPV)-negative tumors are more painful than HPV-positive ones (57). Regarding anatomical location, the pain associated with bone cancer has a specific feature. When tumor cells proliferate in the bone marrow, which is a space devoid of elasticity, this growth inevitably leads to physical constraints on other cell types within this microenvironment. This can result in various consequences, including anemia, bone fractures, and pain. Bone cancer is particularly significant because many metastatic cancers, regardless of the location of the primary tumor, tend to metastasize in the bone marrow for several reasons. One reason is the high vascularity of the bone marrow (58). Another one is the expression of CXCR4 by many metastatic tumor cells and by normal hematopoietic cells trapped in the bone marrow by this specific chemoreceptor (59).

Finally, TME is capable of generating factors that can sensitize nociceptors, called algogenic mediators, or, conversely, possess analgesic properties (29). The production of such mediators likely varies from one tumor type to another and, consequently, the balance of pro- or anti-nociceptive factors as well. For instance, in the early stages following a sunburn, keratinocytes can produce endogenous opioid peptide β-endorphin, which produces analgesia (60). It can be speculated that such a process could explain, for example, the relatively painless nature of melanoma. Thus, the TME of different cancers may contain different sets of algogenic and anti-nociceptive factors, able to either increase or dampen painful stimuli.

*How to manage cancer pain?* Cancer pain has a significant impact on the quality of life and mental health of patients. Consequently, it is crucial to tailor analgesic treatments based on the specific type of pain experienced by cancer patients. The primary strategy for managing cancer pain has traditionally followed the World Health Organization’s (WHO) guidelines for cancer pain relief. This strategy involves matching the potency of analgesia with the severity of pain, employing a range from basic analgesics to powerful opioids. Consequently, paracetamol and non-steroidal anti-inflammatory drugs (NSAIDs) are employed to address mild pain in patients, whereas mild and potent opioids such as codeine, tramadol, and morphine are utilized for the management of moderate to severe pain (61, 62). Accurately defining the type of cancer pain that a patient is experiencing is crucial, as it may necessitate the implementation of additional strategies like adjuvant analgesia (using antiepileptic or antidepressant drugs), corticosteroids, radiotherapy, and interventional procedures (63). For instance, neuropathic pain can be partially treated by combining opioids with antiepileptic or antidepressant drugs (64, 65). While these medications improve the quality of life for patients with cancer pain, their side effects are significant and require careful management during the treatment period (66).

### 2.3 Molecular and cellular mechanisms of pain in cancer

As mentioned earlier, the molecular and cellular mechanisms underlying cancer pain are numerous and occur at various levels of the pain circuit (**Figure 1**). In this review, our primary focus will be on the molecular and cellular mechanisms that take place within the tumor microenvironment (TME) or its vicinity. While we acknowledge the importance of neuropathic pain in cancer, we will not delve extensively into it due to space constraints. Nor will we discuss the mechanism of pain cancer regulation at the CNS level (termed central sensitization), which has been well documented elsewhere (31).

#### 2.3.1 Algogenic mediators within the TME

**Figure 2:**
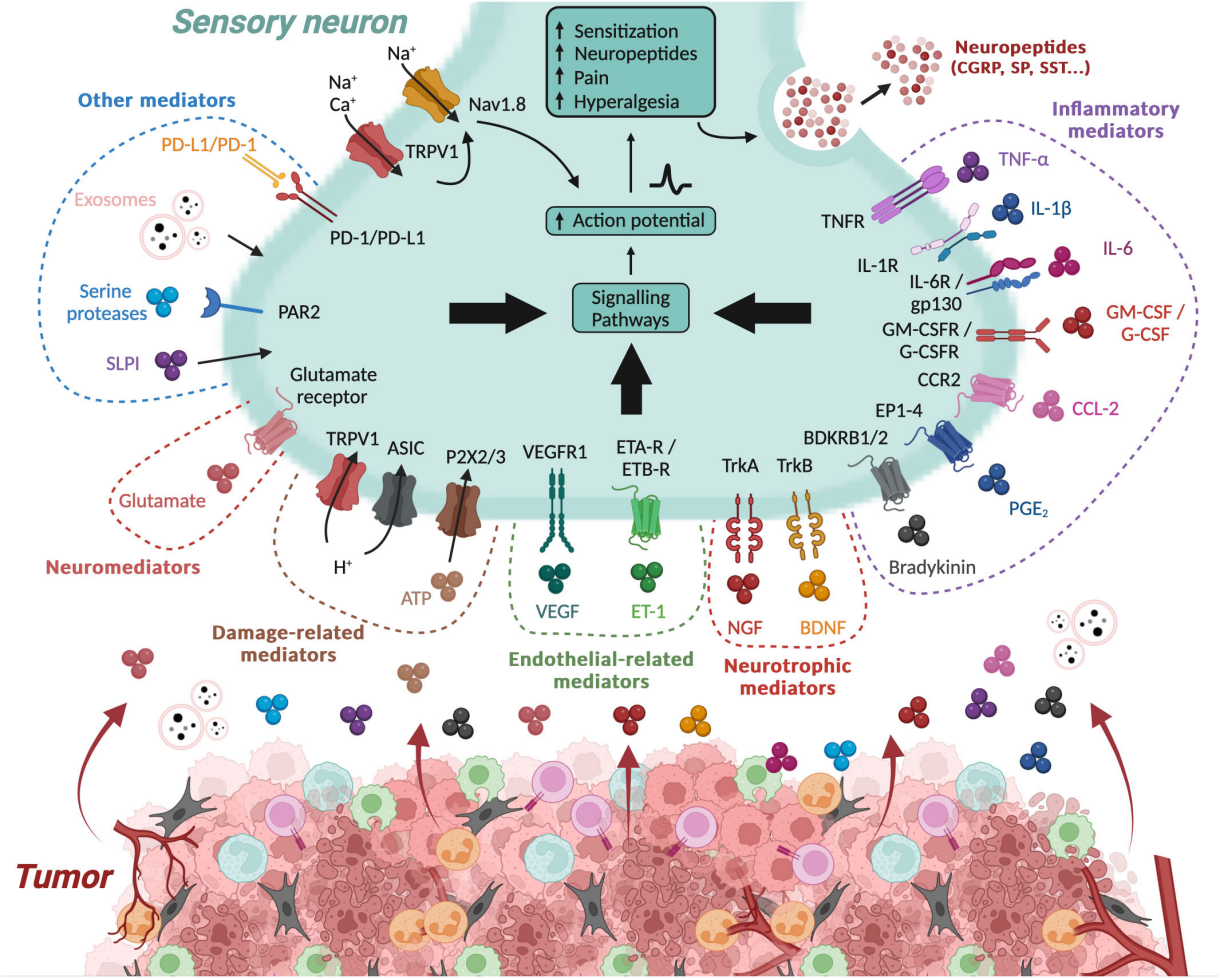
Algogenic factors in the tumor microenvironment. The tumor microenvironment (TME) generates numerous algogenic factors that are capable of sensitizing the peripheral sensory neurons (PSNs) innervating the tumor. This sensitization initiates a signaling cascade in the PSN, leading to sensations of pain or hyperalgesia, and also triggers the release of neuropeptides such as CGRP and SP. Some of these factors are associated to inflammation within the tumor, such as cytokines and chemokines, while other molecular factors are directly linked to tumor cells, such as exosomes. Inflammation and hypoxic conditions also promote tumor cell necrosis, leading to the production of damage-associated molecular patterns (DAMPs). Additionally, several other mediators, such as neurotrophic factors, neuromediators, and angiogenesis-related molecules, possess algogenic properties. On the contrary, certain molecule like PD-L1 can inhibit the activity of PSNs by limiting the activation of TRPV1. ASIC, acid-sensing ion channel; ATP, adenosine triphosphate; BDKRB1/2, bradykinin receptor B1/2; BDNF, brain-derived neurotrophic factor; cAMP, cyclic adenosine monophosphate; CGRP, calcitonin gene-related peptide; CCL-2, C–C motif chemokine 2; CCR2, C–C chemokine receptor type 2; EP1–4, prostaglandin E2 receptor 1-4; ETA-R, endothelin receptor type A; ETB-R, endothelin receptor type B; ET-1, endothelin 1; G-CSF, granulocyte colony-stimulating factor; G-SCFR, granulocyte colony-stimulating factor receptor; GM-CSF, granulocyte-macrophage colony-stimulating factor; GM-CSFR, granulocyte-macrophage colony-stimulating factor receptor; Gp130, glycoprotein 130; IL, interleukin; NGF, nerve growth factor; PAR2, proteinase-activated receptor 2; PD-1, programmed death 1; PD-L1, programmed death-ligand 1; PGE2, prostaglandin E2; P2X2/3, purinergic receptor P2X2/3; SLPI, secretory leukocyte protease inhibitor; SP, substance P; TNFα, tumor necrosis factor alpha; TNFR, tumor necrosis factor receptor; Trk, tropomyosin receptor kinase; TRPV1, transient receptor potential cation channel subfamily V member 1; VEGF, vascular endothelial growth factor; VEGFR1, vascular endothelial growth factor receptor 1. Created with BioRender.com.

One of the major mechanisms underlying cancer pain is the production and release of algogenic mediators within the TME. These mediators, either through their unique ligands or via other receptors and second messenger systems, can sensitize PSN, thereby playing a role in the development of hyperalgesia and allodynia within the TME (31, 46, 67) (**Figure 2**).

The algogenic mediators can originate from a variety of sources within the TME, including tumor cells and immune cells, and encompass a diverse range of molecules such as inflammatory factors, cytokines, chemokines, colony-stimulating factors, and neurotrophic factors (**Figure 2**). Environmental changes (extracellular ATP and proton rises) can also lead to the generation of such mediators. These mediators have multiple mechanisms of action. Some interact directly with receptors or ion channels involved in the detection and signaling of noxious stimuli, such as protons by TRPV1 and acid-sensing ion channel (ASIC) receptors (68–72). Others induce sensitization of these receptors/channels. For example, they can induce the activation of various kinases, including protein kinase A, protein kinase C, and c-Src kinase, capable of phosphorylating TRPV1, leading to its sensitization (73–77). They can also increase the expression of these receptors/channels (78, 79). For example, by binding to its TrkA receptor, nerve growth factor (NGF) induces heightened expression of TRPV1 and ASIC3, and neurotransmitters such as SP, CGRP, brain-derived neurotrophic factor (BDNF), and various sodium and calcium channels that regulate nociceptor excitability (79). In addition, TRPV1 sensitization can result from functional interactions with other receptors, such as P2X3 or NMDA (80, 81). The list of algogenic mediators is too extensive to be exhaustively presented here. However, in **Table 1**, we have referenced a list of those known to play a role in cancer pain.

**Table 1:** Algogenic mediators involved in cancer pain.

Mediators	Cancer type	Involved receptors	Ref
Inflammatory mediators			
TNF-α	Oral cancer (mouse)	TNFR (1 or 2)	(82)
	Bone cancer (rat)	TNFR1	(83, 84)
	Squamous cell carcinoma (mouse)	TNFR2	(85)
	Lung cancer (human)	n.d.	(86)
IL-1β	Bone cancer (mouse/rat)	IL-1R	(83, 84, 87)
	Lung cancer (human)	n.d.	(86)
IL-6	Bone cancer (rat)	IL-6R	(78, 83, 84)
	Squamous cell carcinoma (mouse)	gp130	(88)
	Lung cancer (human)	n.d.	(86)
G-CSF/GM-CSF	Bone cancer (mouse/rat)	G-CSFR/GM-CSFR	(89, 90)
CCL2	Fibrosarcoma (mouse)	n.d.	(91)
PGE2	Bone cancer (mouse)	n.d.	(92)
Bradykinin	Bone cancer (mouse)	BDKRB1	(93)
	Melanoma (mouse)	BDKRB1/2	(94)
Neurotrophic mediators			
NGF	Oral cancer (mouse/human)	TrkA	(43)
	Bone cancer (mouse)		(55, 95–98)
	Bone cancer (human)		(98)
BDNF	Oral cancer (mouse)	TrkB	(99, 100)
Others mediators			
Endothelin-1	Oral cancer (rat)	ETA-R/ETB-R	(101, 102)
	Bone cancer (mouse)		(103, 104)
Serine protease	Oral cancer (mouse/human)	PAR2	(105–107)
	Squamous cell carcinoma (mouse)		(108)
SLPI	Melanoma (mouse)	n.d.	(109)
ATP	Squamous cell carcinoma (mouse/human)	P2X2/3	(110)
	Bone cancer (mouse/rat)		(111–116)
Proton	Bone cancer (rat)	ASICs	(68)
	Multiple myeloma (mouse)	ASIC3	(69)
	Bone cancer (mouse)	Trpv1	(70)
Exosomes	Oral cancer (mouse/human)	Multiple	(117)
Glutamate	Bone cancer (rat)	NMDA1, mGluR7, mGluR8	(118)
VEGF	Bone cancer (mouse)	VEGFR1	(119)
	Pancreatic cancer (mouse)		
	Squamous cell carcinoma (mouse)		

ASIC, acid-sensing ion channel; ATP, adenosine triphosphate; BDKRB1/2, bradykinin receptor B1/2; BDNF, brain-derived neurotrophic factor; CCL-2, C–C motif chemokine 2; ETA-R, endothelin receptor type A; ETB-R, endothelin receptor type B; ET-1, endothelin 1; G-CSF, granulocyte colony-stimulating factor; G-SCFR, granulocyte colony-stimulating factor receptor; GM-CSF, granulocyte-macrophage colony-stimulating factor; GM-CSFR, granulocyte-macrophage colony-stimulating factor receptor; GluR, glutamate receptor; Gp130, glycoprotein 130; IL, interleukin; n.d., not determined; NGF, nerve growth factor; NMDA1, N-methyl-d-aspartate-type glutamate receptors; PAR2, proteinase-activated receptor 2; PGE2, prostaglandin E2; P2X2/3, purinergic receptor P2X2/3; SLPI, secretory leukocyte protease inhibitor; TNF-α, tumor necrosis factor alpha; TNFR, tumor necrosis factor receptor; Trk, tropomyosin receptor kinase; Trpv1: transient receptor potential cation channel subfamily V member 1; VEGF, vascular endothelial growth factor; VEGFR1, vascular endothelial growth factor receptor 1.

Regarding inflammatory mediators, PSNs express receptors for many of them, including cytokine receptors (TNFR, IL-1βR, IL-6R, and IL-17RA) and G-protein-coupled receptors for histamine, and prostanglandin E2 (PGE2) (**Figure 2**). The activation of these receptors is known to increase the excitability of nociceptive neurons and to enhance sensitization to subsequent stimuli, thus actively contributing to this process (120). The involvement of these mediators is well-documented in neuropathic pain, where the recruitment of pro-inflammatory cells like macrophages and T cells in the DRG and spinal cord has been evidenced (121–128). The importance of these immune cells and their pro-inflammatory cytokines in the maintenance of chronic pain is supported by experiments in which their depletion significantly reduced existing pain (123, 127, 128). Inflammatory mediators are not only produced by immune cells but also by glial cells like astrocytes and microglia. For instance, following peripheral nerve injury, neurons release chemokines such as CCL2 and CX3CL1 (129), along with other immune mediators like colony-stimulating factor 1 (CSF-1) and ATP (130, 131). These molecules can strongly activate spinal cord glia, leading to the release of pro-inflammatory cytokines (e.g., TNF-α, IL-1β, and IL-6), chemokines (e.g., CCL2), and nitric oxyde (NO) (132).

In the context of cancer pain, one must take into account components of TME, i.e., immune cells, which can produce substantial quantities of inflammatory algogenic mediators, and thus contribute to pain development and/or maintenance. The involvement of certain pro-inflammatory cytokines like TNF-α (82–86, 133), IL-1β (83, 86, 87, 133, 134), and IL-6 (78, 83, 86, 88) and other inflammatory mediators like PGE2 (133) in cancer pain has been reported in various cancers. Consequently, various NSAIDs are frequently used in clinical practice as adjuncts to stronger analgesics to potentially provide additional pain relief. For instance, in bone cancer where inflammatory mediators significantly stimulate afferent PSNs, NSAIDs are commonly used to alleviate cancer pain (135). However, clinical evidence supporting a substantial analgesic effect of classical NSAIDs like selective COX-1 and COX-2 inhibitors in cancer pain is generally lacking (136). Interestingly, drugs targeting pro-inflammatory molecules such as anti-TNF-α and anti-IL-1β are emerging as highly promising options for pain control and could potentially be employed in managing cancer pain (137).

In contrast to pro-nociceptive algogenic mediators, some factors present anti-nociceptive properties. One example is PD1/PD-L1 molecules (for programmed cell death protein 1/programmed death-ligand 1), when expressed on PSNs (138–141). The administration of PD-L1 to naive mice induces analgesia by activating PD-1 and downstream SHP-1 phosphorylation, leading to inhibition of the function of sodium channels and TRPV1, and enhancing the function of potassium channels (TREK2) in PSNs (138, 139, 142). In the context of cancer, the blockade of the PD-1/PD-L1 axis, either through anti-PD-1 administration or SHP-1 deletion in nociceptors, has been shown to aggravate cancer pain in mouse models of melanoma and bone cancer (138, 139). Moreover, in a recent study, Wanderley et al. demonstrated that combining anti-PD-L1 with chemotherapy can exacerbate chemotherapy-induced neuropathic pain (143). However, it is important to note that in a metastatic bone cancer model, despite a temporary increase in pain sensitivity after each treatment, anti-PD-1 immunotherapy leads to long-term advantages by preventing bone destruction and relieving bone cancer pain through the suppression of osteoclastogenesis (144). All these results have obviously many implications for the use of anti-PD1/anti-PD-L1 as immune checkpoint inhibitors and underscore their potential adverse effects on cancer pain. Other anti-nociceptive factors, such as endogenous opioids (enkephalins and endorphins), may also be secreted within the TME (145, 146). These molecules, typically released by immune cells during inflammation, interact with opioid receptors on the surface of nerve endings of sensory neurons to reduce pain sensitization (147). In a mouse model of oral cancer, tumor-infiltrating neutrophils have been shown to produce β-endorphins, thus alleviating tumor-associated pain (148). Paradoxically, in mammary tumor mice model, the presence of β-endorphins within the TME have a pro-nociceptive effect while promoting tumor development (149).

#### 2.3.2 Ectopic nerve sprouting

In addition to nociceptor sensitization through algogenic mediators, cancer-related pain can also arise from heightened tissue innervation resulting from ectopic nerve sprouting (150)(**Figure 1**). Within the TME, various neurotrophic factors such as NGF, BDNF, glial cell–derived neurotrophic factor (GDNF), and vascular endothelial growth factor (VEGF) are released, leading to nerve sprouting (54–56, 97, 119, 151–155). Furthermore, it has been demonstrated that proteins involved in axonal guidance, such as molecules from the ephrin and netrin families, play a role in nerve sprouting within tumors. For instance, Netrin-1 induces the sprouting of sensory nerves and exacerbates pain sensitivity (156, 157). Additionally, Madeo et al. have shown that tumor cells can release the necessary molecules for axonogenesis, particularly ephrin family molecules, through exosome secretion (158). This disorganized sprouting results in a general increase in nerve fiber density, encompassing both sensory and sympathetic nerve fibers and the formation of neuroma-like structures. These changes contribute to episodes of severe acute pain and, in some cases, pain triggered by movement (54). In a healthy bone, sensory and sympathetic fibers are typically segregated. However, tumor-induced sprouting disrupts this separation, allowing sympathetic fibers to potentially activate nociceptive stimuli, thereby exciting adjacent sensory fibers (54).

#### 2.3.3 Perineural invasion and neuritis

PNI is a process in which tumor cells infiltrate neighboring nerves (159). This process facilitates metastatic dissemination and nerve compression and promotes pain induction (**Figure 1**). Clinically, PNI is a recognized characteristic in cancer, often linked to a grim prognosis across various cancer types (160). PNI is prevalent in numerous malignant tumors, such as pancreatic cancer, oral cancer, gastric carcinoma, and biliary tract tumors. Interestingly, cancers that are notorious for causing pain tend to exhibit high rates of PNI. For instance, pancreatic cancer, known for its severe pain, frequently presents with PNI at rates ranging from 80% to 100% correlating with diminished survival and reduced quality of life (161, 162). PNI significantly contributes to the pain experienced by cancer patients, primarily due to the overlap between signaling molecules involved in PNI, such as neurotrophins and chemokines, and those involved in pain signaling (163). Furthermore, tumor cell invasion can lead to neuropathic pain by damaging the neural sheath, rendering nerve processes susceptible to detrimental extracellular matrix stimuli. Additionally, the communication between pancreatic cancer cells and nerves results in abnormal nerve growth and enlargement (48, 153).

In addition to tumor cell invasion, nearby nerves may also be infiltrated by immune cells, resulting in neuritis and neuropathic pain (164) (**Figure 1**). For example, in human pancreatic ductal adenocarcinoma (PDAC), pancreatic nerves were observed to be infiltrated by different types of leukocytes, and the presence of mast cells was strongly correlated with the sensation of abdominal pain in PDAC (165).

### 2.4 Cancer pain and psychological stress

In addition to pain, it is essential to consider that cancer also induces significant psychological stress. This stress primarily operates through stress hormones, namely, (nor-)adrenaline and glucocorticoids, which are released due to the activation of the sympathetic nervous system and the hypothalamic–pituitary–adrenal (HPA) axis (166). The influence of psychological stress and stress hormones on tumor development and progression has been extensively demonstrated and reviewed elsewhere (166). As a whole, the sympathetic nervous system is generally considered to promote tumorigenesis, notably by inhibiting CD8^+^ T cells priming and favoring their exhaustion (167, 168). Nevertheless, in the case of PDAC, it has been suggested that sympathetic neurons may exert an anti-tumor effect (152). Concerning glucocorticoids, recent studies have revealed that local glucocorticoid production can drive the dysfunctional or exhausted state of CD8^+^ T cells, undermining therapy-induced antitumor immunity and the response to immune checkpoint inhibitors (169, 170).

**Figure 3:**
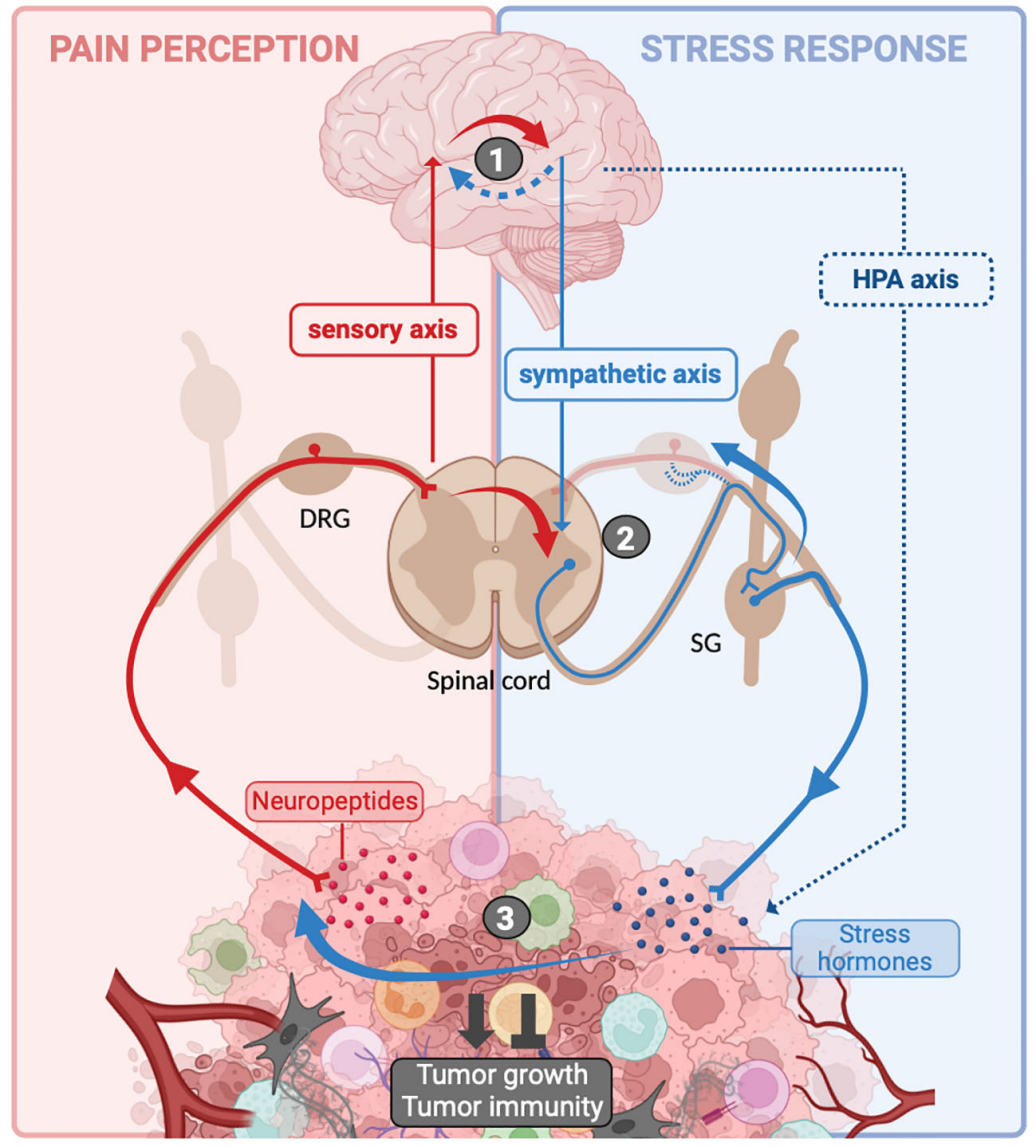
Crosstalk between pain and stress response in cancer. Sensitization of peripheral sensory neurons (PSNs) within the tumor microenvironment (TME) leads to the localized release of neuropeptides. Additionally, these neurons transmit an afferent nerve influx to the spinal cord, which relays this information to the brain. The cognitive processing of this information in the brain is responsible for pain perception (background). In the meantime, in response to the psychological stress caused by the disease, the brain activates the hypothalamic–pituitary–adrenal (HPA) axis and sympathetic neurons, resulting in the release of stress hormones [(nor-)adrenaline and glucocorticoids] into the TME (blue background). Interactions between the sensory axis and the sympathetic/HPA axis can take place at several levels: (1) at the brain level, afferent nerve impulses can trigger a stress response, resulting in activation of the HPA axis and the sympathetic system (solid red arrow). Similarly, it is possible that the stress induced by the disease may enhance pain perception (blue dotted arrow). (2) At the spinal cord and dorsal root ganglia (DRG) level, PSNs can transmit nerve impulses to interneurons that directly activate sympathetic neurons (solid red arrow). Additionally, due to nerve injury, sympathetic neurons from sympathetic ganglia (SG) may extend into DRG to activate PSNs. (3) Within the tumor itself, stress hormones may sensitize PSNs (solid blue arrow). All of these interactions result in the differential integration of pain and stress, and they influence tumor growth and the associated anti-tumor immune response. Created with BioRender.com.

Surprisingly, there is a limited understanding of the connection between pain and stress, despite their frequent co-occurrence in cancer patients (**Figure 3**). However, it appears that the stress response, notably involving the sympathetic nervous system, plays a role in regulating cancer-related pain. For instance, the blockade of the sympathetic nervous system is a common approach to managing abdominal pain in cancer, although the precise underlying cellular and molecular mechanisms remain incompletely elucidated (171). In the context of oral cancer, specifically squamous cell carcinoma of the head and neck, recent research has demonstrated that the release of noradrenaline by sympathetic neurons stimulates the production of TNF-α by cancer cells. This TNF-α can then activate PSNs, leading to the experience of painful sensations (172). In addition to noradrenaline, sympathetic neurons secrete other molecules, including neuropeptide Y (NPY) and adenosine triphosphate (ATP), which can act on postganglionic sympathetic neurons expressing their specific receptors (173, 174). NPY has been implicated in both pro- and anti-nociceptive effects (173). Regarding ATP, it is stored and released along with noradrenaline in the synaptic vesicles of postganglionic sympathetic nerves (175). During stress-induced noradrenaline release, this ATP co-release could act on purinergic receptors expressed by PSNs and sensitized them. Additionally, considering that cancer induces neuropathic pain, it is plausible that post-ganglionic sympathetic neurons may sprout into DRG to activate PSNs and coordinate painful stimuli, as observed in nerve injury models (176). Finally, another compelling argument supporting the influence of stress on pain modulation is the fact that PSNs also express adrenergic receptors, which have the capacity to sensitize these neurons (172, 177–180).

Conversely, PSNs have been shown to promote efferent sympathetic nervous system feedback or HPA activation following stimulation of PSNs in a model of systemic inflammation (181) and atherosclerosis (182). The activity of the sympathetic neurons is regulated by descending supraspinal and primary afferent inputs. Indeed, some brain regions such as the rostral ventroLateral medulla (RVLM) and the paraventricular nucleus activate inhibitory or excitatory interneurons to modulate the activity of preganglionic sympathetic neurons (183). In addition to these descending inputs from the brain, afferent PSNs can also directly influence the activity of pre-ganglionic sympathetic neurons through the activation of propriospinal interneurons (**Figure 3**). This notably occurs after spinal nerve injury, where PSNs can overstimulate interneurons independently of supraspinal control. This results in the excessive activation of sympathetic neurons, leading to the dysfunction of multiple organs (184, 185). Finally, activation of PSNs can also attenuate sympathetic tone, as demonstrated in the bone marrow to regulate bone metabolism (186–188).

Collectively, there is therefore a body of evidence suggesting the existence of a potentially vicious cycle involving pain, stress, and tumor progression, warranting further investigation.

## 3 Cancer, sensory neurons and tumor immunity

### 3.1 Sensory innervation of the tumor

The peripheral nervous system plays a crucial role in maintaining the balance of healthy tissues by coordinating various molecular, cellular, and organic functions. Likewise, the homeostasis of tumors (like that of any organ) orchestrates these processes to optimize their functioning (189). Increasing evidence indicates that, just like in healthy tissues, tumors also modulate their innervation levels to optimize their growth and survival (190).

The observation that tumors can be highly innervated is not recent, and in a publication dating from 1897, Hugh H. Young already observed the presence of nerves in 50% of tumors (191). Despite this initial observation and the fact that as early as the mid-twentieth century, it had been shown that denervation could have an effect on tumor growth (192), the presence of nerve fibers in tumors and their influence on tumor growth remained controversial until the 2000s (193–195). Since then, numerous studies have clearly shown that human and murine tumors are extensively innervated by sympathetic and parasympathetic neurons. Although less well documented, sensory innervation of tumors has also been revealed in certain types of tumors (**Table 2**).

**Table 2:** Sensory innervation in human and mouse tumors.

Cancer type	Human		Mouse	
	Sensory neuronal marker	Ref	Sensory neuronal marker	Ref
Breast	TRPV1	(196)	CGRP	(197)
Head and Neck	TRPV1	(158)	TRPV1	(158)
	CGRP	(198)	CGRP	(198)
Parathyroid	SP, (TH)	(199)	n.d.	n.d.
Skin	TRPV1	(109)	Nav1.8	(109, 200, 201)
Ovary	TRPV1	(202)	TRPV1	(203)
Uterus	TRPV1, VIP, (TH)	(204)	n.d.	n.d

CGRP, calcitonin gene-related peptide; n.d., not determined; SP, substance P; TH, tyrosine hydroxylase; TRPV1, transient receptor potential cation channel subfamily V member 1; VIP, vasoactive intestinal peptide.

Several studies have indicated that tumor tissues from melanoma and ovarian cancer patients exhibit increased sensory innervation marked by TRPV1^+^ neurons when compared to healthy tissues (109, 203). As mentioned above, this could be the result of ectopic nerve sprouting. Additionally, various sensory neuronal markers in cancer patients have shown associations with different prognosis. The level of sensory innervation and the expression of sensory neuronal markers like TRPV1 and Nav1.8 have been linked to poor prognosis in breast cancer (196). In other cancer types, such as ovarian cancer, oral cancer, and melanoma, the relationship appears to be less clear, with some studies suggesting a favorable prognosis (200, 201, 205), while others indicate an unfavorable one (109, 198, 202, 206, 207). Furthermore, research has underscored the diagnostic significance of CGRP levels across various cancer types (208–212). In the case of SP, findings from patient samples data suggest that elevated SP levels are associated with poorly differentiated tumors in oral squamous cell carcinoma and gastric tumors (213, 214).

### 3.2 Influence of sensory neurons on cancer growth

**Figure 4:**
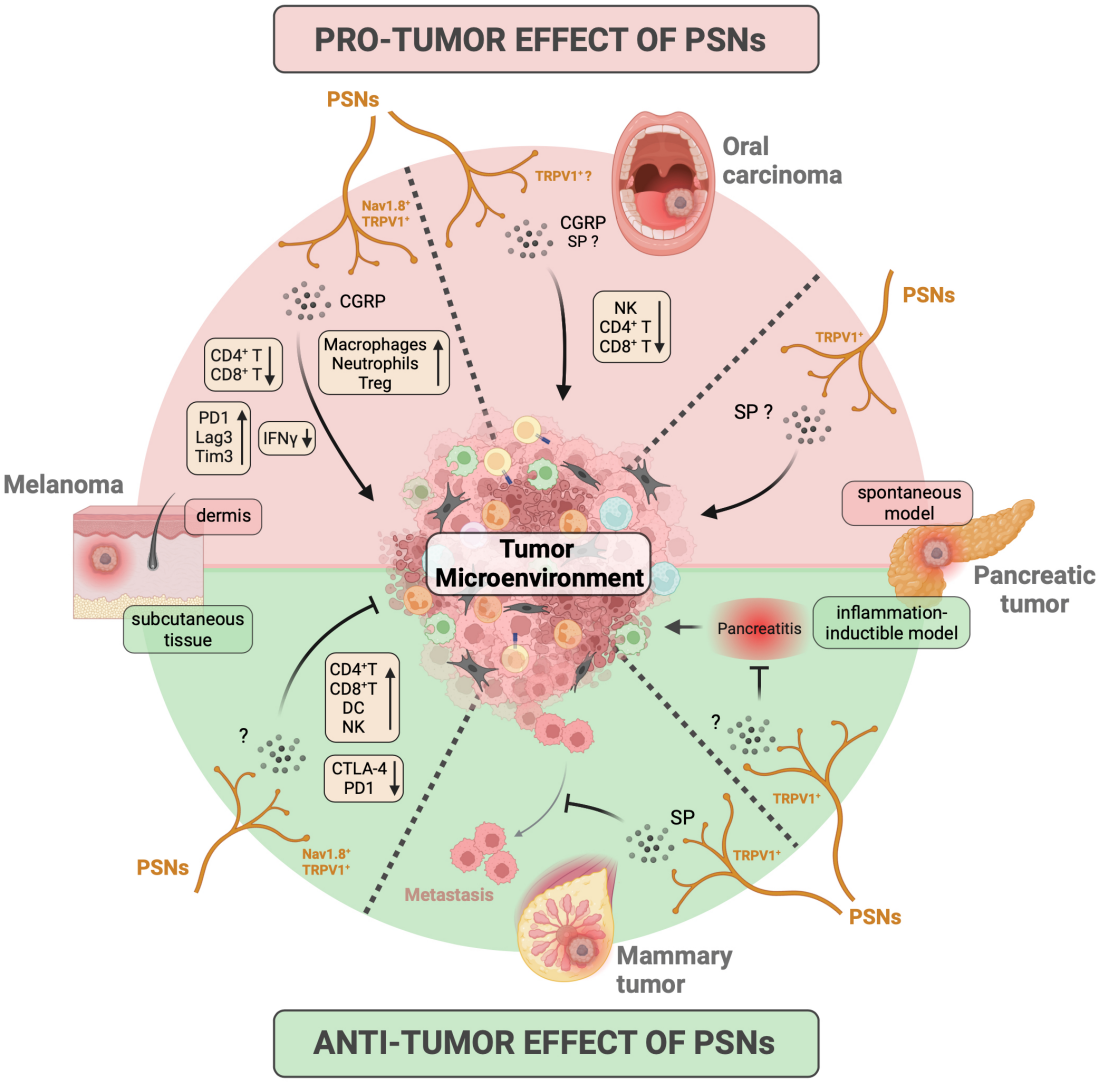
Influence of peripheral sensory neurons on tumor growth and antitumor immune response. The impact of peripheral sensory neurons (PSNs) on cancer development has primarily been investigated in murine models of melanoma, oral squamous carcinoma, pancreatic cancer, and breast cancer/metastasis. In these models, PSNs expressing Nav1.8 and TRPV1 ion channels release the neuropeptides CGRP and SP into the tumor microenvironment (TME). This leads to either pro-tumoral effects (indicated by the red background at the top) or anti-tumoral effects (indicated by the green background at the bottom) on primary tumors or metastases, depending on the specific cancer models studied. The results obtained for each tumor/model are represented in separate quadrants, delineated by dashed lines. Specifically, PSNs promote the growth of primary tumors in oral carcinoma models and inhibit the ability of mammary tumors to metastasize. Their influence on melanoma and pancreatic cancer is mixed and can depend on factors such as the location of melanoma tumor cell injection (pro-tumoral when injected in dermis and anti-tumoral when injected subcutaneously) and the genetic models of pancreatic cancer used (pro-tumoral in spontaneous development models and anti-tumoral in an inflammatory inducible model). Furthermore, PSNs also influence the anti-tumor immune response, affecting factors such as leukocyte recruitment, cytokine production, and the expression of exhaustion markers on T cells (as indicated in the yellow box). CGRP, calcitonin gene-related peptide; CTLA-4, cytotoxic T-lymphocyte antigen-4; DC, dendritic cell; IFN, interferon; NK, natural killer lymphocyte; PD1, programmed cell death protein 1; SP, substance P; Tim3, T-cell immunoglobulin and mucin containing protein-3; Treg, regulatory T cell; TRPV1, transient receptor potential cation channel subfamily V member 1. Created with BioRender.com.

While the impact of sympathetic and parasympathetic fibers on tumor growth has been extensively studied and well-documented (18), the role of sensory fibers, in particular, nociceptors, has only recently gained attention (**Table 3**, **Figure 4**).

**Table 3:** Influence of peripheral sensory neurons on tumor growth and antitumor immune response in tumor mouse models.

Type of tumor	Tumor model	Neuronal modulation model	Neuronal subset involved	Neuromediator involved	Effect of sensory modulation on		Overall effect of sensory neurons	Ref
					Tumor growth/metastasis number	Tumor immunity		
Mammary tumor	4T1 cells injection (mammary pad)	Depletion of TRPV1^+^ neurons (high-dose capsaicin treatment)	TRPV1^+^ neurons	n.d.	Increased metastasis number	n.d.	Anti-tumoral	(215)
	4T1 cells injection (mammary pad)	Depletion of TRPV1^+^ neurons (high-dose capsaicin treatment)	TRPV1^+^ neurons	SP (?)	Increased metastasis number	n.d.	Anti-tumoral	(216)
	4TBM cells injection (mammary pad) + Radiotherapy	SP injection	n.d.	SP	Decreased tumor growth	Decreased infiltration of MDSC	Anti-tumoral	(217)
						Increased infiltration of Treg		
					Decreased metastasis number	Increased production of IL-6 and IFNγ		
						Decreased production of TNFα, IL-10, and MIP-2		
	4TBM injection (mammary pad)	NK1R antagonist treatment	n.d.	SP	Increased metastasis number	Decreased infiltration of MDSC	Anti-tumoral	(218)
						Increased infiltration of CD4^+^ and CD8^+^ T cells		
						Increased production of IL-17		
	4TLM injection (mammary pad)	NK1R antagonist treatment	n.d.	SP	Decreased metastasis number	Increased infiltration of MDSC	Pro-tumoral	(218)
						Increased production of IL-6, TNFα and IL-17		
	4TBM cells injection (mammary pad)	TRPV1^+^ neuron activation (olvanil treatment)	TRPV1^+^ neurons	SP (?)	Decreased metastasis number	Increased infiltration of CD4^+^ and CD8^+^ T cells	Anti-tumoral	(219)
						Increased production of IFNγ, IL-1β and IL-10		
	Human MDA-MB-231 cells injection (mammary pad) in immunodeficient mice	DRG neuron injection	All sensory neurons	n.d.	Increased metastasis number	n.d.	Pro-tumoral	(196)
Oral carcinoma	Human Pci-13 cells injection (tongue) in immunodeficient mice	Surgical ablation of lingual nerve	All sensory neurons	Noradrenaline	Decreased tumor growth	n.d.	Pro-tumoral	(220)
			(Parasympathetic neurons?)					
	MOC1 and MOC2 cells injection (tongue)	Depletion of CGRP (Calca^−/−^ mice)	n.d.	CGRP	Decreased tumor growth	Decreased infiltration of NK cells, CD4^+^ and CD8^+^ T cells	Pro-tumoral	(198)
	MOC7 and mEERL cells injection (oral cavity)	Depletion of TRPV1^+^ neuron (TRPV1Cre-DTAflox mice)	TRPV1^+^ neurons	SP	Decreased tumor growth	n.d.	Pro-tumoral	(221)
		NK1R antagonist treatment						
Melanoma	B16 cells injection (s.c.)	DRG neuron injection	All sensory neurons	n.d.	Increased tumor growth	Increased infiltration of MDSC	Pro-tumoral	(222)
	B16F10 cells injection (s.c.)	Depletion Nav1.8^+^ neurons (Nav1.8Cre-DTAflox mice) Depletion of TRPV1^+^ neurons (RTX treatment)	Nav1.8^+^ TRPV1^+^ neurons	n.d.	Increased tumor growth	n.d.	Anti-tumoral	(201)
	B16F10 cells injection (s.c.)	Inhibition of Nav1.8^+^ neurons (Nav1.8Cre-hM4Di mice)	Nav1.8^+^ neurons	n.d.	*Inhibition*: increased tumor growth	*Inhibition:* Decreased infiltration of IL-17^+^CD4^+^ T cells and CD8^+^ T cells	Anti-tumoral	(200)
		Activation of Nav1.8^+^ neurons (Nav1.8Cre-hM3Dq mice)			*Activation*: decreased tumor growth	*Activation:* Increased infiltration of DC, NK, γδT cells, CD4^+^ and CD8^+^ T cells Decreased infiltration of neutrophil Increased percentage of IL-17^+^CD4^+^ Decreased expression of CTLA-4 and PD1 on T cells		
	B16F10 cells injection (i.d.)	Depletion of TRPV1^+^ neurons (RTX treatment) Surgical ablation of thoracic cutaneous nerve	TRPV1^+^ neurons	n.d.	Decreased tumor growth	Increased infiltration of CD11b+ cells, B cells, CD4^+^ and CD8^+^ T cells Decreased infiltration of Treg, CD206^+^ macrophages, neutrophil Decreased expression of PD1 and CD73 on T cells Increased percentage of IFNγ^+^- and GzmB^+^- CD8^+^ T cells Increased number of tertiary lymphoid structure Increased of B and T cells repertoires	Pro-tumoral	(206)
	B16F10, Yummer1.7 and Yummer cells injection (i.d.)	Depletion of Nav1.8^+^ neurons (Nav1.8Cre-DTAflox mice) Activation of Nav1.8^+^ neurons (Nav1.8Cre-ChR2 mice) Depletion TRPV1^+^ neurons (TRPV1Cre-DTAflox mice) Inhibition of TRPV1^+^ neurons (QX314 treatment) Depletion of Ramp1 (Ramp1^−/−^ mice) Ramp1 antagonist treatment	Nav1.8^+^ TRPV1^+^ neurons	CGRP	*Depletion/Inhibition*: decreased tumor growth	*Depletion:* Increased infiltration of CD8^+^ T cells Increased % of IFNγ^+^ CD8^+^ T cells Increased CD8^+^ T cells cytotoxicity Decreased % of exhausted PD1^+^Lag3^+^Tim3^+^CD8^+^ T cells	Pro-tumoral	(109)
					*Activation*: increased tumor growth			
Pancreatic tumor	Genetic model of PDAC: LSL-KrasG12D, p53Lox, p48Cre (KPC) mice	Depletion of TRPV1^+^ neurons (high-dose capsaicin treatment)	TRPV1^+^ neurons	n.d.	Decreased PanIN lesion number	n.d.	Pro-tumoral	(223)
	Genetic model of PDAC: LSL-KrasG12D, LSL-Trp53R172H, Pdx1Cre (KPC) mice	Depletion of TRPV1^+^ neurons (Resiniferatoxin treatment)	TRPV1^+^ neurons	SP (?)	Decreased PanIN lesion number	n.d.	Pro-tumoral	(224)
	Genetic model of PDAC susceptibility inductible by pancreatitis: LSL-KrasG12D, Pdx1Cre (KC) mice + cerulein treatment	Activation of TRPV1^+^ neurons (low-dose capsaicin containing diet)	TRPV1^+^ neurons	n.d.	Decreased PanIN lesion number	n.d.	Anti-tumoral	(225)

CGRP, calcitonin gene-related peptide; CTLA-4, cytotoxic T-lymphocyte antigen-4; DC, dendritic cell; GzmB, granzyme B; i.d., intradermously; IFN, interferon; IL, Interleukin; LAG3, lymphocyte-activation gene 3; MDSC, myeloid-derived suppressor cell; MIP, macrophage inflammatory proteins; n.d., not determined; NK, natural killer lymphocyte; PD1, programmed cell death protein 1; PDAC, pancreatic ductal adenocarcinoma; RTX, resiniferatoxin; SP, substance P; s.c., subcutaneously; Tim3, T-cell immunoglobulin and mucin containing protein-3; Treg, regulatory T cell; TRPV1, transient receptor potential cation channel subfamily V member 1.

The initial studies highlighting the impact of nociceptors on tumor development date back to the early 2000s, using mouse models of orthotopic 4T1 mammary tumors via chemical denervation experiments induced by administration of a high dose of capsaicin, a specific ligand of the TRPV1 receptor. Erin et al. first demonstrated that TRPV1^+^ PSNs do not affect the growth of primary mammary tumors but inhibit the formation of cardiac and pulmonary metastases, via a mechanism involving SP and its receptor neurokinin-1 receptor (NK-1R) (215, 216). However, they also showed that, conversely, NK1R antagonists could reduce the number of metastases when injecting a highly aggressive metastatic breast cancer cell line, suggesting a detrimental effect of SP in this case (218). Furthermore, in another model involving mammary tumor xenografts, it was demonstrated that co-administration of murine DRG neurons with human MDA-MB-231 cells in the orthotopic setting promoted pulmonary metastasis (196). In this case, *in vitro* experiments established a connection between the release of Semaphorin 5A by PSNs and tumor cell migration via Plexin B3 expression. Thus, in breast cancer, it appears that nociceptors primarily impact the metastatic process. Moreover, it is difficult to definitively determine the nature of their influence, whether it is beneficial or detrimental, as it seems to depend strongly on the model used and the degree of aggressiveness of the tumor cells.

In murine PDAC models, chemical denervation of TRPV1^+^ nociceptors leads to a decrease in the number of pancreatic intraepithelial neoplasms (PanIN) and PDAC lesions, resulting in improved mouse survival (223, 224). Co-culture experiments between DRG neurons and PDAC organoids have revealed that SP induces the secretion of trophic factors by neuroendocrine cells within neoplastic acini, thereby fostering tumor growth (224). Conversely, in a model stimulating PanIN lesion development through the induction of chronic pancreatitis, Bai et al. demonstrated that TRPV1^+^ nociceptor activation by diet supplemented with low-dose capsaicin exerts anti-tumor effects, leading to reduced PanIN damage and decreased expression of molecules associated with tumor cell proliferation (225). These discrepancies are likely due to differences in the models employed to induce PanIN/PDAC. Notably, in the pancreatitis-induced neoplasia model (225), the observed effects could be due to the control of inflammation by nociceptors rather than to its impact on carcinogenesis.

In oral cancer, tumor growth is diminished in mice lacking TRPV1^+^ PSNs (221) or CGRP (198) in orthotopic oral carcinoma mouse models. This effect might be attributed to the presence of receptors for CGRP (RAMP1 for receptor activity modifying protein 1) and SP (NK-1R) on tumor cells (198, 221) and/or immune cells (198). In another oral squamous cell carcinoma model induced by the carcinogen 4-nitroquinoline 1-oxyde (4NQO), Amit et al. have revealed that trigeminal PSNs undergo reprogramming into sympathetic-like neurons upon the integration of extracellular vesicles released by tumor cells (220). In this study, the release of noradrenaline by these reprogrammed PSNs fosters tumor growth, and the surgical removal of the lingual nerve leads to reduced tumor growth (220). Note, however, that the lingual nerve is not exclusively composed of PSNs but also contains parasympathetic fibers from the facial nerve (226), introducing a potential bias in the interpretation of these results. Hence, in the case of oral cancer, it appears that nociceptors may have a detrimental effect by promoting tumor growth.

Finally, studies using mouse models of melanoma have produced contradictory results. While some studies have indicated a pro-tumoral effect of Nav1.8^+^ and TRPV1^+^ PSNs (109, 206), others have reported the opposite (200, 201). They all used the B16 cell line as a melanoma model, but differ in the way the cells were injected. In experiments demonstrating pro-tumor effects, tumor cells are injected intradermally (109, 206), whereas those showing anti-tumor effects employ subcutaneous injection (200, 201). The different skin layers (epidermis, dermis, and subcutaneous tissue) exhibit distinct innervation and immune cell composition, the intradermic injection being the most immunogenic (227). This information is particularly significant, as Balood et al. have shown that the influence of nociceptors seems to depend on the degree of immunogenicity of the tumor cells used (109). Therefore, these anatomic and topological differences could account for these divergent outcomes. In addition, given that melanoma originates from malignant melanocytes located in the basal lamina of the epidermis, intradermal injection seems to correspond more to physiological conditions.

Due to the paucity of studies and to key experimental differences, one cannot definitively conclude that PSNs have a pro- or anti-tumor role. In addition to experimental differences, such as those observed in melanoma models, it seems that the impact of sensory fibers, harmful or beneficial, likely depends on the specific type of tumor and its anatomical location.

The observed impact of nociceptors on tumor growth might be a result of their influence on different cell types within the TME. For instance, one of the first known functions of neuropeptides is their capacity to regulate inflammation by directly acting on endothelial cells and smooth muscles, thereby controlling vascular permeability, extravasation, and blood flow (228). These vascular effects may impact tumor development by potentially influencing aspects such as the supply of nutrients and oxygen, or the infiltration of immune cells into the tumor. Other effects might also be attributed to the impact of nociceptors on tumor cells. Indeed, several studies have demonstrated that neuropeptide receptors are expressed on tumor cells, and their binding to neuropeptide ligands regulates various processes, including proliferation, survival, adhesion, and migration. This is particularly noteworthy, which have been shown to be involved in the progression of various cancer types, as shown in gastric, breast, and oral cancers, namely, through binding of SP to NK-1R expressed by tumor cells (213, 221, 229–242). Thus, sensory innervation could affect tumor growth not only by acting on tumor cells but also on tumor vascularization. This latter action could depend not only on neuropeptides but also on a structural factor, i.e., the fact that in most organs, the nervous and the vascular tree develop together. In addition, the effects of nociceptors on tumor growth may also result from their capacity to modulate tumor immunity, as discussed below.

### 3.3 Influence of sensory neurons on anti-tumor immunity

In addition to studying the effect of nociceptors on tumor growth, some studies have evaluated their influence on anti-tumor immunity (**Table 3**, **Figure 4**). As expected, these studies have observed a correlation between the influence of PSNs on tumor immunity and their effects on tumor growth.

In instances where PSNs exhibit a pro-tumor activity, they also tend to negatively affect the anti-tumor immune response (109, 198, 206). For example, in a mouse model of melanoma induced by intradermal injection of B16 cells, the depletion of nociceptors has been shown to increase the presence of immune cells with anti-tumoral properties, including CD4^+^ and CD8^+^ T cells, while reducing the abundance of pro-tumoral cells like regulatory T cells (Treg), tumor-associated macrophages (TAM), and neutrophils (109, 206). Moreover, in the absence of nociceptors, tumor-infiltrating CD8^+^ T lymphocytes (CD8^+^ TILs) display enhanced cytotoxicity; produce granzyme B, IFNγ, and TNF-α more effectively; and exhibit reduced exhaustion, as indicated by the lower expression of immune-checkpoint molecules PD-1, T-cell Immunoglobulin domain and mucin domain 3 (TIM-3), and lymphocyte activation gene 3 (LAG3). Balood et al. have shown that this is the result of a direct effect of CGRP on CD8^+^ TILs expressing RAMP1, the CGRP receptor, on their surface (109).. In this model, CGRP production by nociceptors is triggered by secretory leukocyte protease inhibitor (SLPI) released by B16 melanoma tumor cells. Vats et al. have also shown that PSNs prevent the formation of tertiary lymphoid structures (TLS), which play a role in the development of an effective anti-tumor melanoma response (206). Additionally, in an oral squamous cell carcinoma model, the depletion of CGRP leads to an increase in the infiltration of naturel killer (NK) and T cells (198). Thus, there is a whole set of studies in which nociceptors exert a pro-tumoral effect by inhibiting the anti-tumoral immune responses.

In contrast, in others studies, anti-tumor effects of nociceptors on tumor growth have been reported, mediated by an impact on anti-tumor immunity (200, 217, 219). In a melanoma mouse model induced by subcutaneous injection of B16 cells, activating nociceptors has been found to enhance the recruitment of NK, CD4^+^, and CD8^+^ T cells, which exhibit a less exhausted phenotype with reduced PD1 and cytotoxic T-lymphocyte-associated protein 4 (CTLA-4) expression (200). As for tumor growth, discrepancies in melanoma studies may be attributed to injection methods, either intradermal or subcutaneous. Additionally, in a model of metastatic breast cancer cell line injection, inhibiting the NK-1R receptor resulted in a decreased recruitment of CD4^+^ and CD8^+^ T cells and increased recruitment of myeloid-derived suppressor cells (MDSC) in metastasis (218). In the same model, stimulating nociceptors with TRPV1 agonist led to an enhanced recruitment of CD4^+^ and CD8^+^ T cells, along with improved cytokine production (243). However, it is worth noting that when a less aggressive metastatic breast cancer cell line was used, the authors observed the opposite outcome (218). Lastly, they also demonstrated that SP improved radiotherapy treatment efficacy by increasing IFN-γ and IL-6 production while reducing IL-10 production by restimulated leukocytes and by decreasing MDSC infiltration (217).

### 3.4 Putative effects of nociceptor mediators on tumor-infiltrating immune cells

In addition to these few studies, the putative effect of sensory neurons on the anti-tumor immune response can be apprehended with regard to the effects already described of nociceptor mediators, mainly CGRP and SP neuropeptides, on immune cells in a non-cancer context and/or within experiments carried out *in vitro*.

In most scenarios, SP boosts the immune response by increasing the release of inflammatory cytokines in the acute phase and enhancing the cytotoxicity of immune effectors. It improves mouse T-cell activation and IFN-γ production (244–246) and stimulates human T-cell proliferation by enhancing IL-2 production (247–249). SP may also promote the function of effector CD8^+^ T cells in autoimmune disorder, such has alopecia areata (250). It regulates neutrophil influx and enhances their phagocytic activity in inflamed tissues (251). SP potentiates the immunostimulatory role of skin-resident Langerhans cells, leading to the activation of T helper 1 (Th1)and IFN-γ-producing CD8^+^ T cells (252). However, in response to allergens in the skin, it acts also on dermal dendritic cells (DCs), promoting their migration to the draining lymph nodes (LNs), where they induce Th2 activation (253). SP may also elevate IL-12 production by DCs and macrophages (254–256). SP can additionally boost monocyte chemotaxis (257) and stimulate their release of pro-inflammatory cytokines, thus promoting Th17 cell generation (258). In a dose-dependent manner, SP increases NK cell migration and IFN-γ secretion (259, 260). SP also facilitates the generation of memory CD8^+^ T cells during the primary immune response by improving antigen-presenting cells (APCs) function (248). In specific conditions, it counteracts the immunosuppressive activity of Treg (261). Thus, SP may be viewed as a nerve-released, pleiotropic potentiator of the activation of pro-inflammatory immune cells.

Concerning the effects of CGRP on immune cells, these effects appear to be more contrasted than those of SP. On the one hand, a significant number of studies has pointed to its primarily immunosuppressive characteristics. For instance, CGRP suppresses the production of IFN-γ, TNF-α, and IL-2 by CD4^+^ T cells (262–265). Treatment of Langerhans cells with CGRP has also been shown to reduce their ability to migrate to draining LNs (266), to stimulate T-cell proliferation (267, 268), and to promote the production of IFN-γ, CXCL9, and CXCL10 by CD4^+^ T cells (269). Moreover, treatment of human monocytes or DCs with CGRP inhibits their functions and significantly decreases the proliferative response of allogeneic T cells (270–272). These effects may be attributed to CGRP downregulating the expression of class II major histocompatibility complex (MHC-II) and CD86 co-stimulatory molecules and inhibiting the release of IL-12, IL-1β, TNF-α, and CCL4 by APCs (268, 271–274). In the case of infection by *Staphylococcus aureus* in the lungs or *Streptococcus pyogenes* in the skin, CGRP also inhibits the recruitment of neutrophils to the affected tissues (10, 11). Lastly, CGRP plays a crucial role as an inhibitory factor for type 2 innate lymphoid cell (ILC2) responses, restraining acute airway inflammation (275, 276). In contrast, studies have shown that CGRP may have an immuno-activating effect. For instance, several studies across different models suggest that CGRP promotes type 17 immune response. First, it has been observed that CGRP exacerbates experimental autoimmune encephalomyelitis by promoting Th17 functions (277). In addition, in psoriasis and *C. elegans* infection models, CGRP stimulates DCs to produce IL-23 thereby facilitating the inappropriate activation of IL-17-producing γδT cells (5, 9). Finally, in a model of optogenetic activation to TRPV1^+^ nociceptors, induction of type 17 inflammation requires the vesicle release of CGRP (278). Recently, Hanc et al. conducted a detailed investigation into the effects of CGRP on DC biology, revealing that DC activation in the presence of nociceptors enhances their production of pro-inflammatory cytokines. Moreover, CGRP induces a heightened sentinel phenotype in DCs, characterized by increased pro-IL-1β in CGRP-treated DCs (279). To account for these seemingly contradictory results, we propose the following hypothesis: CGRP may interact with different types of target cells in the body, and these cells may exhibit different inflammatory responses. For example, CGRP may act on immune cells to decrease the production of proinflammatory cytokines, but it may also act on endothelial cells to promote vasodilation and increase inflammation under certain conditions. Moreover, the co-release of other peptides by PSNs producing CGRP, depending on the stimulus/challenge, could also account for some of theses contradictory results.

We have mainly focused on CGRP and SP here, but it is worth noting that PSNs release a whole range of other neuropeptides and factors that can influence immune cell responses. For example, vasoactive intestinal peptide (VIP) is involved in promoting allergic inflammation in asthma by acting on ILC2 and Th2 cells (6). In addition, PSN-derived TAFA-4 plays a role in facilitating tissue repair by modulating the inflammatory profile of macrophages (13). Finally, PSNs also have the ability to generate chemokines, including CCL2, which contribute to immune cell recruitment (122, 222, 279, 280).

All of these findings, mainly observed in non-cancer context or *in vitro* studies, emphasize the capacity of neuropeptides and, consequently, nociceptors to enhance or suppress cytotoxic responses. This could potentially impact the anti-tumor immune responses.

### 3.5 Transcriptional changes of PSNs induced by the tumor

As described above, PSNs can be sensitized by the tumor through algogenic mediators or neuronal lesions leading to cancer pain (**Figure 2**). The TME can also induce structural changes, such as growth and axon branching in PSNs, included in the ectopic neuronal sprouting evoked earlier (109, 197, 220, 222). Furthermore, tumor cells can induce a metabolic change in PSNs, leading to a reduced oxygen consumption and a deficit in neuronal electrical activity in a colon cancer model (281).

In addition, it is likely that tumors trigger profound transcriptional changes in the PSNs that innervate them. PNI, tumor compression causing neuronal damage, and chronic hypersensitivity of neurons innervating the tumor could be responsible for these transcriptomic changes. Such a phenomenon has been described after nerve injury, such as sciatic nerve ligation or transection (20). In this study, nerve injury induced transcriptomic changes leading to transient loss of identity of sensory neuron subsets in DRGs. This was associated with increased expression of nerve injury or activation markers such as the transcription factor Atf3 (activating transcription factor 3) and Sox11 (SRY-Box transcription factor 11). Interestingly, a similar induction of Atf3 is also observed in PSNs innervating the tumor. Thus, an increase in Atf3 expression has been shown in the PDAC model (45, 223) and in various bone cancer models (282–284). In the case of pancreatic cancer, this could be the consequence of its particular ability to induce PNI, causing neuronal damage and neuropathic pain (162). Regarding oral cancer, in a xenograft model, no Atf3 expression was observed (99), in contrast to a syngeneic model (285). However, even in the absence of Atf3 expression in PSNs, the tumor can induce a significant transcriptional change in the tumor-innervating PSNs, leading to increased transcription of multiple genes, including some involved in nociceptor sensitization (99, 281, 286). Furthermore, in an oral squamous cell carcinoma model, it was observed that trigeminal PSNs undergo reprogramming into sympathetic-like neurons upon the integration of extracellular vesicles released by tumor cells (220). Finally, in *in vitro* co-culture experiments, Ballod et al. have also shown that DRG neurons exposure to melanoma cells leads to changes in their transcriptome (109).

As done in nerve injury models (20), it is crucial to thoroughly document the transcriptomic changes induced by the tumor in PSNs and investigate its impact on tumor development and the associated immune response.

## 4 Key research questions

What we have discussed in this review paves the way for numerous questions and future research endeavors.

- Pain, resulting from the activation of nociceptors and the infiltration of the immune system both seem to carry prognostic significance in the context of cancer progression. Is there a connection between these two factors, and does a triple correlation exist between the pain level of a tumor, the extent of immune cell infiltration, and its progression?
- Considering that nociceptors may potentially exert a beneficial influence on the anti-tumoral immune response, it would be important to assess whether the use of analgesic treatments targeting these nociceptors (e.g., lidocaine) could lead to detrimental suppression of antitumor immunity.
- To date, most research into the effect of PSNs on the anti-tumor immune response has focused primarily on peptidergic nociceptors, specifically the Nav1.8^+^ TRPV1^+^ PSNs. Nevertheless, recent findings indicate that other types of PSNs, particularly non-peptidergic nociceptors, play a role in modulating inflammation and the immune response (13, 14). Could these PSNs also be involved in regulating the anti-tumor immune response?
- Current studies on the effects of nociceptors on tumor growth and immune response primarily evaluate their action through the local release of neuropeptides. However, one of their main features is to generate a signal that leads to an efferent response involving other types of neurons (e.g., sympathetic and parasympathetic) that can also influence tumor growth and immunity. This aspect of their influence, and their interactions with the different branches of the nervous system, is still largely unexplored and should be the focus of future investigations.
- The relationship between pain and psychological stress and how they influence each other is still relatively poorly documented. An intriguing question is whether the stress generated by the disease could promote cognitive sensations of pain. Given its prevalence and their significance for the well-being of patients, it is essential to gain a better understanding of the molecular and cellular mechanisms that underlie the relationship between pain and psychological stress.

## 5 Concluding remarks

Cancer pain is typically regarded as a byproduct of the disease, and it is managed as such. However, it also reflects the activation of PSNs, particularly nociceptors, which may have important consequences for cancer development, tumor immunity, and thus cancer treatment. Thus, in addition to the cognitive perception of pain, the recognition of noxious signals results in the activation of PSNs leading to the peripheral or central production of neuromediators, such as neuropeptides. These neuromediators have the capacity to directly impact not only tumor cells but also all cells within TME, including immune cells engaged in anti-tumor immune responses. They can modulate these cell functions, either positively or negatively. Conversely, the tumor itself exerts influence on PSNs via physical mechanisms (e.g., nerve compression or injury), PNI, or the release of algogenic mediators. This can result in hypersensitization or damage to these neurons, contributing to the generation of cancer-related pain, and potentially leading to significant deep transcriptomic alterations.

In summary, there exists a complex interplay between the tumor and the sensory nerve fibers within it. This interplay can take on either a detrimental or beneficial nature, depending on the specific characteristics of the cancer, and it undeniably has profound implications for tumor development and associated immunity. Beyond the fundamental interest in understanding the influence of PSNs on tumor growth and anti-tumor immunity, future research could lead to the discovery of new anti-cancer therapeutic targets, such as neuropeptides and their receptors, that could eventually be combined with other anti-cancer treatments, such as chemotherapy or immunotherapy, to improve their efficacy.

## Author contributions

UM: Conceptualization, Writing – original draft, Writing – review & editing. NB: Conceptualization, Writing – original draft, Writing – review & editing. CD: Conceptualization, Writing – review & editing. VF: Conceptualization, Funding acquisition, Project administration, Supervision, Writing – original draft, Writing – review & editing.
